# Can *Bacillus thuringiensis* Toxin and Sticky Traps Be Used in Integrated Control of *Trialeurodes vaporariorum* in Greenhouses?

**DOI:** 10.3390/plants15162511

**Published:** 2026-08-20

**Authors:** Guan-Bing Liu, Bo-Wen Liang, Tao Wang

**Affiliations:** Ministry of Education Key Laboratory for Ecology of Tropical Islands, Key Laboratory of Tropical Animal and Plant Ecology of Hainan Province, College of Life Sciences, Hainan Normal University, Haikou 571158, China

**Keywords:** *Trialeurodes vaporariorum*, phototaxis, yellow sticky traps, *Bacillus thuringiensis*

## Abstract

The greenhouse whitefly (*Trialeurodes vaporariorum*) causes substantial yield losses and reduced market value of produce in protected agricultural systems worldwide. This study examined the biocontrol potential of recombinant *Bacillus thuringiensis* (Bt) toxin proteins harboring the LBW08 gene. Within a decision-support system (DSS) framework, we evaluated the effects of different competent cell lines on toxin expression profiles and assessed the insecticidal performance of two Bt toxin variants (08 and 08bt), both individually and in combination with yellow sticky traps. Our goal was to generate quantitative parameters on population suppression dynamics and cost-effectiveness, providing a basis for DSS-driven pest management recommendations. The 08Bt toxin expressed in JM110 competent cells exhibited superior purity and consistent insecticidal activity against *T. vaporariorum* nymphs. Notably, the integrated application of 08Bt and yellow sticky traps achieved the highest level of population suppression among all treatments, highest numerical level of population suppression among all treatments, although pairwise differences were not statistically significant, suggesting strong potential for synergistic control. In contrast, no synergistic interaction was observed with the 08 variant. However, the 08 variant combined with traps did not show a comparable numerical improvement. These findings support the development of cost-effective, environmentally sound tactics that can be incorporated into broader IPM strategies for protected agriculture.

## 1. Introduction

*Bacillus thuringiensis* (Bt) insecticidal proteins are highly specific toward lepidopteran, coleopteran, and dipteran pests. They have been widely adopted as environmentally safe alternatives to synthetic pesticides [1,2]. The classification of Cry proteins provided a theoretical basis for understanding this specificity [3]. Subsequent studies revealed that Bt has evolved specific toxins to target different insect orders [4]. The structure, diversity, and evolution of Bt toxins have been extensively reviewed [5], and their biocidal activity against a wide range of insect orders has been comprehensively documented [6]. Elucidating the mode of action of Bt toxins and optimizing application parameters can guide targeted pest suppression [7].

The greenhouse whitefly, *Trialeurodes vaporariorum*, is a globally prevalent pest in protected agriculture. It infests numerous hosts across the Solanaceae, Fabaceae, Brassicaceae, and Cucurbitaceae families [8,9,10,11,12]. Both adults and nymphs feed on phloem sap and excrete honeydew. This promotes sooty mold growth and impairs photosynthesis, often causing more severe damage than direct feeding [13,14,15]. The species also transmits multiple plant viruses, further reducing crop yield and quality [15]. Its high fecundity, short generation time, and strong adaptability make it exceptionally difficult to control [16,17].

Currently, chemical pesticides remain the primary control method of *T. vaporariorum*. However, prolonged overuse of pesticides has led to pest resistance, environmental contamination, and harm to non-target organisms, contradicting the principles of sustainable agriculture [18]. This situation has created an urgent need for safe and efficient green control technologies [14,17,18].

Although whiteflies feed predominantly on phloem sap, nymphs (especially the sessile stages) probe the leaf surface and epidermal/mesophyll cells extensively during stylet penetration. During these probing events, they may ingest surface-deposited toxins that are suspended in liquid droplets or remain on the leaf cuticle. Several studies have reported insecticidal activity of Cry proteins against hemipteran pests when applied as foliar sprays [19,20]. In our study, the spray protocol (80% applied to the abaxial surface) was specifically designed to maximize nymphal exposure. We acknowledge that the exact route of entry (oral vs. possible cuticular penetration) is not yet fully resolved. However, our study was designed as an efficacy trial under realistic greenhouse conditions to test whether Bt preparations can reduce whitefly populations, regardless of the precise mode of action. Future work using fluorescent tracers or gut dissection would be needed to confirm ingestion.

Research on Bt-based management of whiteflies and other hemipteran pests remains limited and has yielded inconsistent and largely unconvincing results. Bt toxins have long been established as effective control agents against chewing pests, but their applicability to piercing-sucking insects such as whiteflies remains highly questionable. Early trials reported only minor and variable mortality in whitefly nymphs, with low overall efficacy and unclarified underlying mechanisms [7]. While some studies have reported insecticidal activity of certain Bt proteins against select hemipteran species under laboratory conditions, the practical feasibility of foliar-applied Bt toxins for field-level whitefly control has not been rigorously validated. Porcar et al. [19] found that several Cry δ-endotoxins exerted toxic effects on the pea aphid (*Acyrthosiphon pisum*). Chougule et al. [20] successfully retargeted the Cyt2Aa toxin against hemipteran pests, including the green peach aphid and achieved significant mortality via engineered modification. Liu et al. [21] characterized two novel ETX/MTX2-type Bt proteins, Cry64Ba and Cry64Ca, which displayed insecticidal activity against aphids and planthoppers in controlled bioassays. These findings suggest potential activity of Bt proteins against certain hemipterans, but do not confirm that naturally occurring Bt toxins can deliver practical control efficacy against whiteflies via conventional foliar application.

In this study, we used two Bt toxin variants, 08 and 08bt, produced in different expression systems, to evaluate their insecticidal activity against *T. vaporariorum* and their integrated performance with yellow sticky traps. The objectives were: (1) to compare the population suppressive effects of Bt toxins and yellow sticky traps against adult whiteflies; (2) to determine whether combining Bt toxins with yellow sticky traps produces enhanced control relative to trap use alone; and (3) to evaluate the practical feasibility of Bt-based strategies for greenhouse whitefly management. Crucially, we interpret our findings through a Decision Support System (DSS) lens. DSS approaches are essential for managing, rather than merely attempting to control highly prolific pests like *T. vaporariorum* [18,22]. By generating dynamic efficacy data and preliminary cost estimates, we aim to provide a quantitative framework that helps growers select the most cost-effective strategy based on initial pest density, desired control timeline, and economic thresholds. Such DSS-informed tactics are more likely to be adopted as part of a sustainable IPM program because they address the economic and practical realities of greenhouse production. This work aims to provide a scientific basis for green integrated control of greenhouse whitefly, extend the theoretical framework of Bt toxin application against piercing-sucking pests, and contribute to sustainable greenhouse production as a component of area-wide IPM.

## 2. Materials and Methods

### 2.1. Experimental Material

#### 2.1.1. Insects

A laboratory colony of *T. vaporariorum* was established from individuals collected at the Ecological Garden of Hainan Normal University and maintained for more than three generations under controlled conditions (approximately 25 °C, 14:10 (L:D) h photoperiod, light intensity 1000–2000 lx, and 50–80% relative humidity) to eliminate potential confounding effects from field-population genetic heterogeneity. Healthy tomato (*Solanum lycopersicum*) seedlings at the four- to five-true-leaf stage served as host plants and were replaced at regular intervals. For all experiments, only uniformly vigorous adults aged three to five days post-eclosion, with a 1:1 sex ratio, were selected. The rearing and experimental conditions were maintained at 25 ± 1 °C, 60 ± 10% relative humidity, and a 14:10 (L:D) h photoperiod.

#### 2.1.2. Reagents and Instruments

All molecular cloning, protein expression and purity verification experiments (Section 2.2.1, Section 2.2.2, Section 2.2.3, Section 2.2.4 and Section 2.2.5) were conducted in the molecular biology laboratory of Hainan Normal University, while integrated control efficacy trials (Section 2.2.6) were performed in a controlled greenhouse at the same research facility.

Taq DNA polymerase, restriction endonucleases, T4 DNA ligase, and DNA Marker were purchased from Takara Bio Inc. (Dalian, China). Genomic DNA extraction kit, plasmid extraction kit, and DNA gel extraction kit were obtained from Omega Bio-tek (Norcross, GA, USA). Ampicillin, kanamycin, and isopropyl-β-D-thiogalactoside (IPTG) were purchased from Sigma-Aldrich (St. Louis, MO, USA). Other chemical reagents were of analytical grade and purchased from Chengdu Kelong Chemical Reagent Factory (Chengdu, China).

Instruments included a PCR amplifier (Bio-Rad, Hercules, CA, USA), a gel imaging system (Tanon, Shanghai, China), a high-speed refrigerated centrifuge (Eppendorf, Hamburg, Germany), and an ultraviolet-visible spectrophotometer (Shimadzu, Kyoto, Japan). The laboratory was maintained at 25 ± 1 °C and 40–60% relative humidity, with standard lighting for routine operations.

#### 2.1.3. Preparation of Recombinant Bacteria Preparation

The Bt recombinant bacteria containing the LBW08 gene were inoculated into LB liquid medium (supplemented with ampicillin) and cultured with shaking at 28 °C and 200 rpm for 96 h until spores and parasporal crystals were fully formed. Bacterial cells were collected by centrifugation (4 °C, 5000 rpm, 10 min), resuspended in sterile normal saline distilled water, and adjusted to a concentration of 1 × 10^8^ CFU/mL (or a series of concentrations for gradient experiments).

##### Verification of Recombinant Bacteria Purity

SDS-PAGE electrophoresis combined with ImageJ software (version 1.54f) was used for quantitative analysis of toxin protein purity, and the specific steps were as follows: (1) Take 1 mL of bacterial culture, centrifuge at 12,000 rpm for 10 min at 4 °C to collect the precipitate, wash twice with PBS buffer (pH 7.4), add 5 × SDS loading buffer, and denature by boiling in a water bath for 10 min; (2) Perform SDS-PAGE electrophoresis using 12% separating gel and 5% stacking gel (stacking gel voltage: 80 V, separating gel voltage: 120 V, electrophoresis time: 90 min); (3) After electrophoresis, stain with Coomassie Brilliant Blue R-250 for 2 h, and decolorize with decolorizing solution (methanol: glacial acetic acid: water = 4:1:5) until the bands were clear; (4) Gray-scale analysis of the electrophoresis image was performed using ImageJ software, and the purity was calculated as the ratio of the gray value of the target protein band (approximately 79 kDa, corresponding to Cry1Ac protein) to the total gray value of all protein bands.

### 2.2. Experimental Method

#### 2.2.1. Gene Cloning and Expression Vector Construction

(1) Acquisition of LBW08 Gene Sequence and Primer Design

The target gene LBW08 (xpp22Aa-like) was provided by Professor Zhang Wenfei from the Lab of Tropical Island Microbial Resources Development and Utilization. Specific amplification primers were designed using Primer 5.0 software.

Upstream primer P1: 5′-ATGGATAACAATCCGAACACCAATGAATGC-3′ (containing the start codon ATG)

Downstream primer P2: 5′-TTAACCAGTTTGCGGCTGCTGGTG-3′ (containing the stop codon TAA)

(2) PCR Amplification and Gel Extraction

PCR amplification was performed using the plasmid containing the LBW08 gene as the template. The reaction system was as follows (Table 1).

The integrity of the PCR amplicons was assessed via electrophoresis on a 1% (*w*/*v*) agarose gel. Purification of the amplification products was subsequently performed using the SanPrep Column PCR Product Purification Kit (Sangon Biotech, Shanghai, China) in strict accordance with the manufacturer’s protocol. Following purification, the recovered DNA fragments were re-evaluated by 1% agarose gel electrophoresis to confirm purity and size integrity. The thermal cycling program was set as follows (Table 2).

(3) Construction of Expression Vector

A plasmid vector suitable for heterologous expression in *Bacillus thuringiensis* was selected. This vector harbored a compatible replicon for stable maintenance in Bt, an ampicillin resistance cassette for selection, and the strong promoter region derived from the Cry1Ac gene to drive constitutive expression. The pHT315-*cry1Ac* backbone was kindly provided by Professor Wenfei Zhang of the Laboratory of Tropical Island Microbial Resources Development and Utilization. The vector backbone was linearized via PCR amplification employing the thermocycling parameters specified in Section 2.2.1. The resulting amplicons were subsequently purified using the SanPrep Column PCR Product Purification Kit (Sangon Biotech, Shanghai, China) following the manufacturer’s standard protocol. Verification of linearized vector integrity and purity was performed by electrophoresis on a 1% (*w*/*v*) agarose gel. Residual template plasmid DNA was eliminated by digestion with DpnI endonuclease. The digestion reaction system was prepared as described in Table 3.

#### 2.2.2. Seamless Cloning and E.coli Bt Shuttle Expression System

(1) Construction of LBW08bt-PHT315-*cry1AC* recombinant plasmid

Prepare a reaction system on ice and react at 50 °C for 5–30 min (recommended setting is 15 min). After the reaction is complete, place the reaction product on ice for 5 min to terminate the reaction. The reaction products can be directly used for conversion experiments or frozen at −20 °C for future use. The reaction system was prepared as follows (Table 4).

For single fragment ligation: the recommended optimal molar ratio of vector to exogenous insertion fragment is n (vector):n (insertion fragment) = 1:2.2 for recombinant product conversion.

(1) XL10 competent cells were thawed on ice
(2) Take 10 μL of the recombinant reaction product mentioned above and add it to 100 μL of XL10 competent cells. Gently mix and mix well,

Let it sit on ice for 30 min.

(3) After being heated in a 42 °C water bath for 90 s, immediately place it on ice to cool for 2–5 min.
(4) Add 900 μL SOC or LB liquid medium (without antibiotics), 37 °C, 220 rpm

Shake and rejuvenate at rpm for 45 min.

(5) Centrifuge the rejuvenated bacterial solution at 5000 rpm for 2 min, discard the supernatant, and suspend the bacterial body with the remaining culture medium. Use a sterile coating rod to coat the bacterial solution on a plate containing the correct resistance, and invert it in a 37 °C incubator for 12–24 h.
(6) The clones grown on the plate can be quickly identified using the “colony PCR method”.

#### 2.2.3. Positive Clone Identification

(1) Colony/bacterial liquid PCR identification: Use a pipette to pick up a single colony and mix it with 10 μL sterile water, then take 1 L as a template, or directly dip the colony into the PCR system for amplification (it is recommended to use at least one primer located on the carrier to avoid false positive results);
(2) PCR identification using plasmids as templates: Select single colonies into LB medium containing corresponding antibiotics, shake overnight at 37 °C and 220 rpm, extract plasmids as templates, and amplify using vector universal primers or specific primers;
(3) Single colonies were inoculated into LB medium containing the corresponding antibiotics and cultured overnight at 37 °C and 220 rpm. Plasmids were then extracted and digested with the corresponding endonucleases, and fragment sizes were verified by electrophoresis.
(4) Sequencing: Send to Nanshan Biotechnology Company for sequencing comparison.

#### 2.2.4. *E.coli* Bt Shuttle Expression System

Preparation of Bt competent cells, *Bacillus thuringiensis* HD73^−^ single colony was transferred to 10 mL SOB medium and incubated overnight at 28 °C 220R shaking. After transferring the 1% inoculation amount, continue to culture for 2 h, and then add glycine to the final concentration of 2%. After adding glycine, shake at 28 °C for another hour, centrifuge at 1690 g for 10 min to collect the bacterial solution, gently suspend in a tube with 20 mL SG, wash twice with 50 mL SG 3000 g for 5 min, precipitate 4 mL suspension and divide into 100 μL, store at −80 °C for later use. Extract the positive strain LBW08bt-XL10 plasmid, transfer the expression recombinant plasmid into JM110 competent cells, and perform demethylation reaction on the exogenous gene. Extract positive bacterial strains. The LBW08bt-JM110 plasmid was then transformed by electroporation and introduced into the non crystalline protein Bt mutant strain HD73^−^ competent state for expression. The strong promoter of the Cry1Ac gene was selected to express the exogenous gene. For the 08 strain, the same plasmid was transformed directly into HD73^−^ competent cells without the JM110 demethylation step.

#### 2.2.5. Bt Protein Expression

The Bt recombinant bacteria containing the LBW08 gene were inoculated into LB liquid medium (containing ampicillin) and cultured with shaking at 28 °C and 200 rpm for 96 h, until the culture was fully fermented and the protein was abundantly expressed. Bacterial cells were collected by centrifugation (4 °C, 5000 rpm, 10 min), washed twice with PBS buffer, and then disrupted by ultrasonication for 5 min (microtip at 20% power, 10 s on, 10 s off). The supernatant and precipitate were collected separately by centrifugation (4 °C, 12,000 rpm, 15 min). The precipitate was washed twice with 1 mL of PBS buffer, then resuspended in 100 μL of PBS buffer by pipetting.

SDS-PAGE analysis was performed on the protein products (supernatant and precipitate) of the expressed strain and the reference strain. After 12% separation gel electrophoresis, Coomassie Brilliant Blue staining showed that the recombinant strain exhibited a specific target band at approximately 79 kDa, corresponding to the Cry22Aa-like toxin protein. This protein was present mainly as a soluble protein in the supernatant. Quantitative analysis using ImageJ software showed that the gray value of the target protein band accounted for 92.3% of the total protein band gray value, indicating that the expression purity of the toxin protein was high, with no obvious interference from impurities, and could be used for subsequent toxicity testing.

After SDS-PAGE confirmation, the supernatant containing soluble 08/08Bt toxin protein was mixed with the following excipients: corn starch (5 g/L), calcium carbonate (0.5 g/L), gum arabic (10 g/L), and sucrose (10 g/L). The mixture was stirred at room temperature for approximately 3 h, then pre-frozen at −20 °C and lyophilized in a freeze-dryer for 36 h to obtain a powder formulated as an 80% wettable powder (WP). The powder was stored at −20 °C in sealed containers until use. For bioassays, the powder was reconstituted in sterile saline (0.9% NaCl). The total protein concentration was estimated from the optical density (OD_600_) of the bacterial culture prior to processing, and the solution was adjusted to a working concentration of 1.0 mg/mL total protein. Protein purity was verified by SDS-PAGE with ImageJ densitometric analysis, which confirmed that the target toxin band accounted for 92.3% of total protein. Precise protein quantification using the Bradford assay was not performed in this study. Future work should incorporate the Bradford or BCA method to establish exact dose–response relationships.

#### 2.2.6. Greenhouse Trial Design and Data Collection

(1) Experimental design and randomization.

The greenhouse experiment was conducted in a naturally ventilated greenhouse under conditions distinct from those in the laboratory described in Section 2.1.2. The greenhouse was maintained at 25 ± 2 °C, 60 ± 10% relative humidity, and a 14:10 (L:D) photoperiod provided by natural sunlight supplemented with artificial lighting to maintain the photoperiod. The trial was arranged in a randomized complete block design with three blocks conducted over time. Each block contained six treatments: (1) blank control (CK, sprayed with sterile saline only), (2) yellow sticky trap alone, (3) 08 toxin alone, (4) 08Bt toxin alone, (5) 08 toxin + yellow sticky trap, and (6) 08Bt toxin + yellow sticky trap. Within each block, every treatment was applied to six potted tomato plants (one plant per pot), giving a total of 108 experimental units (6 treatments × 6 plants × 3 blocks). Within each block, six potted tomato plants per treatment were grouped together and placed directly on the greenhouse floor. Treatments were separated by spatial arrangement rather than physical netting or screens: adjacent treatment groups within each block were spaced 1 m apart, and replicate blocks were separated by a 1 m unplanted buffer zone to reduce unintended adult whitefly dispersal. This randomization procedure ensures that variation in plant condition, including potential differential pest damage, is distributed across treatment groups, allowing treatment effects to be distinguished from pre-existing plant heterogeneity.

(2) Treatment application.

The freeze-dried protein powder was reconstituted in sterile saline and diluted to a final working concentration of approximately 1.0 mg/mL total protein, with 0.05% (*v*/*v*) Tween-20 added as a surfactant. The solution was applied with a manual sprayer. For each treated plant, 80% of the spray volume was directed to the abaxial leaf surface (the main site of nymphal settlement and feeding), and the remaining 20% was applied to the adaxial surface. A fixed volume of 10 mL per plant was used, spraying until leaves were thoroughly wetted but without runoff. Plants in the CK and trap-alone treatments were sprayed with an equal volume of sterile saline containing 0.05% Tween-20. One yellow sticky trap (25 cm × 20 cm) was hung 10 cm above the canopy of each plant in the trap-containing treatments immediately after spraying. Yellow sticky traps (25 cm × 20 cm, double-sided) were hung 10 cm above the plant canopy immediately after spraying. Traps were replaced every 7 days to maintain adhesive efficacy; at each replacement, all captured insects were removed along with the trap. The number of whiteflies captured on traps was not recorded. Although non-target arthropods (e.g., parasitoids and other small insects) were occasionally observed on the traps, they were neither counted nor identified.

(3) Observations and data collection.

After treatment, all plants were maintained under controlled conditions (25 ± 2 °C, 60 ± 10% RH, 14:10 L:D photoperiod). The number of adult whiteflies on each whole plant and on the yellow sticky trap was counted at 3, 5, 7, 10, 12, 14, 17, 19, and 21 days after treatment. Only adult whiteflies on plants were counted; whiteflies captured on traps were not included in the counts. Nymphs were not systematically counted in this trial.

#### 2.2.7. Data Analysis

All statistical analyses were performed in GraphPad Prism (version 10.1.2) using two-way repeated-measures ANOVA, with treatment and time as factors. This approach enables the separation of treatment effects from temporal trends (including plant condition changes over time), as time is treated as a within-subjects factor. Multiple comparisons were performed using Tukey’s HSD test. Significance was set at *p* < 0.05.

## 3. Results

### 3.1. Cloning, Prokaryotic Expression Vector Construction and Protein Expression of the cry1Ac Gene

#### 3.1.1. Gene Amplification

**Figure 1:**
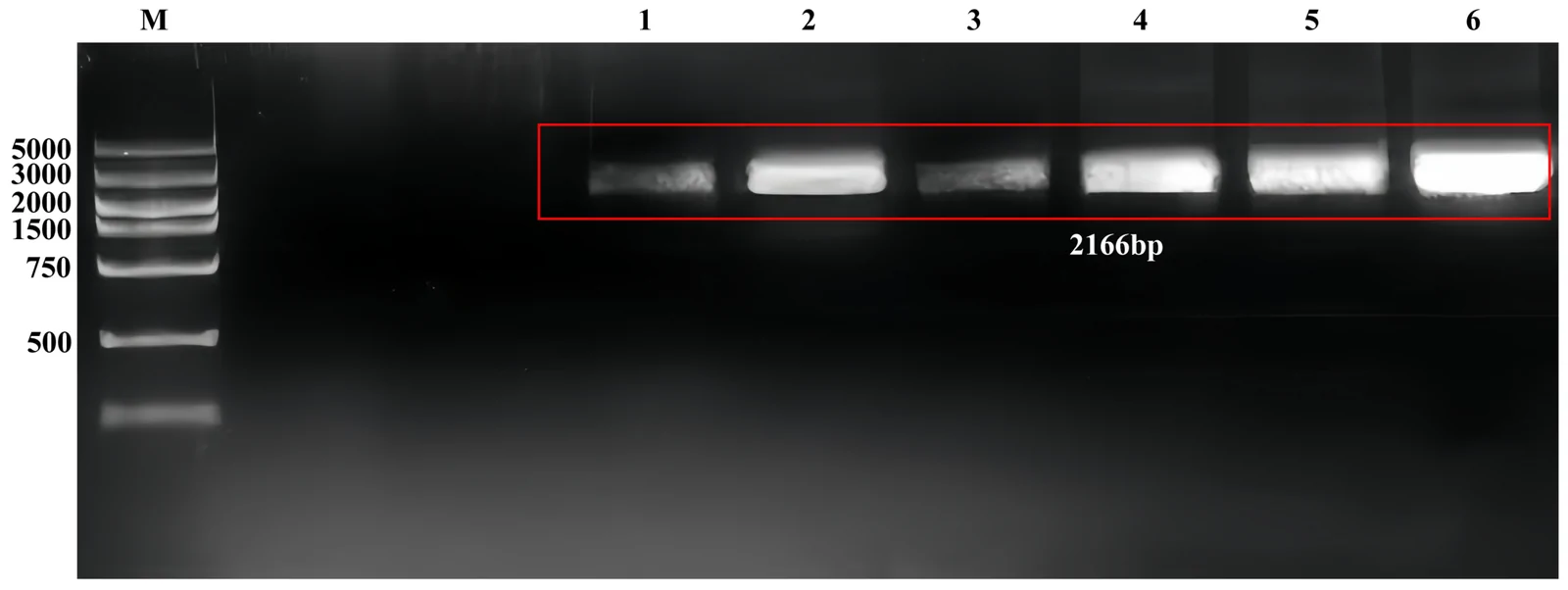
Electrophoresis pattern of PCR amplification products of the cry1Ac gene. The target band with an expected size of 2166 bp is marked with a red box.

PCR amplification of the cry1Ac gene from Bt genomic DNA using primers P1/P2 yielded a single product of approximately 3 kb (Figure 1), consistent with the expected size of the target gene.

#### 3.1.2. Construction and Validation of the Recombinant Expression Vector PHT315-cry1Ac

**Figure 2:**
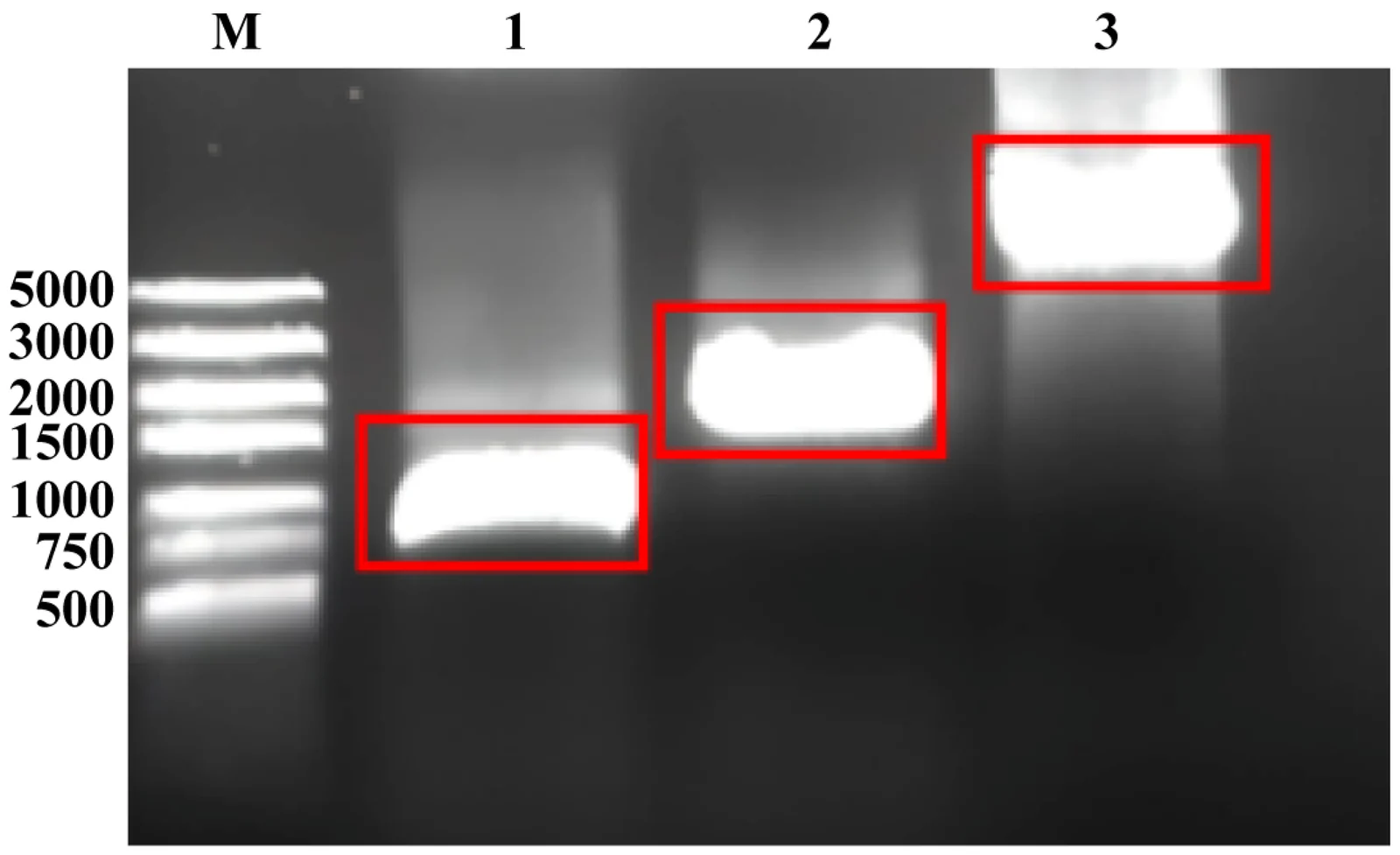
Electrophoresis pattern of the linearized PHT315-cry1Ac vector. The target band corresponding to each lane is marked with red boxes.

**Figure 3:**
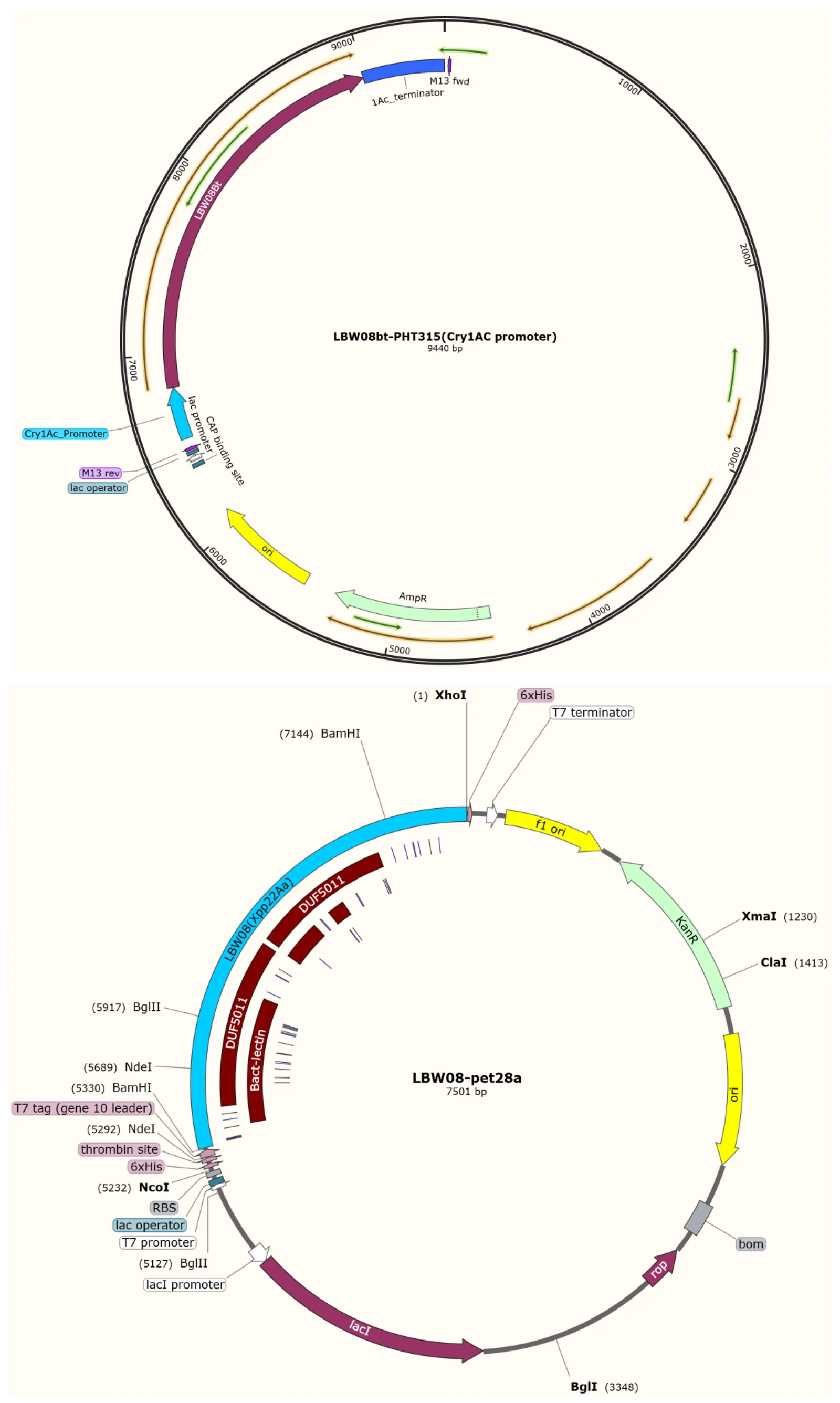
Physical map of the recombinant plasmid LBW08-1ac315. Note: Key elements such as the Cry1Ac promoter, AmpR resistance marker, M13 forward and reverse primer sites, lac operon, and vector backbone are labeled in the figure. The plasmid size is 9440 bp.

**Figure 4:**
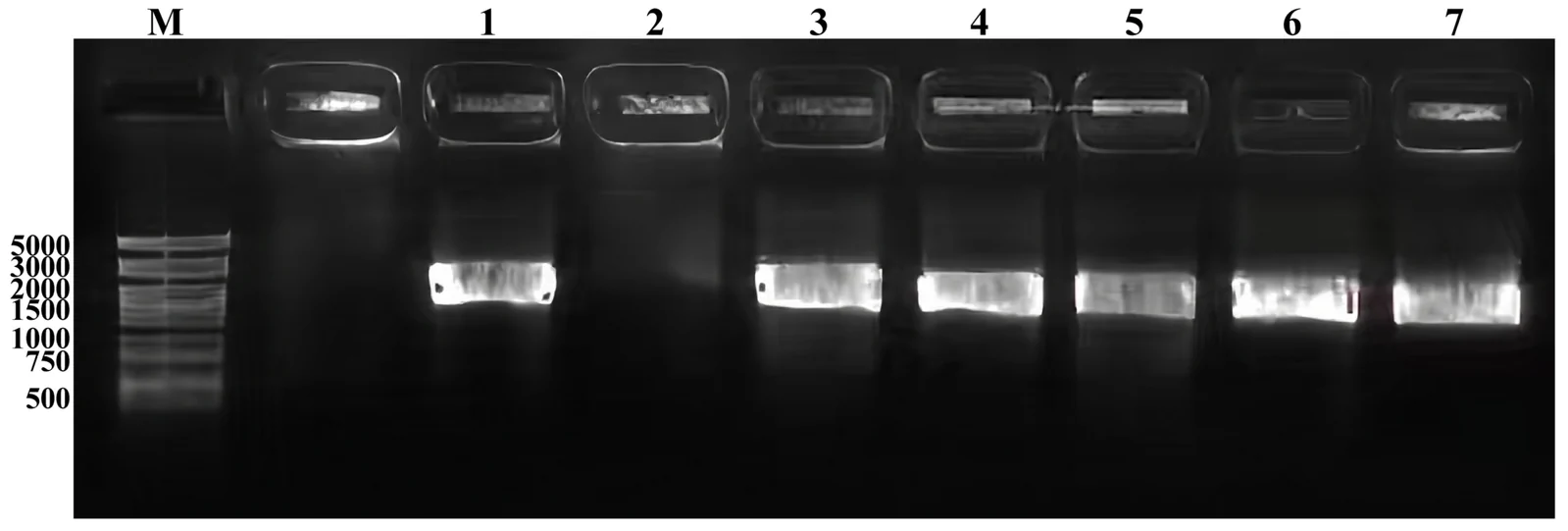
Agarose gel electrophoresis verification of the LBW08-PHT315-cry1Ac recombinant plasmid.

The amplified fragment was inserted into the linearized PHT315-*cry1Ac* vector (Figure 2) via seamless cloning to generate the recombinant plasmid LBW08-1ac315. The complete plasmid was 9440 bp in length (Figure 3). Sanger sequencing confirmed that the inserted sequence was free of mutations or frameshifts. The verified plasmid was subsequently demethylated in *E. coli* JM110, producing high-purity recombinant plasmid suitable for heterologous expression (Figure 4).

#### 3.1.3. Prokaryotic Expression and Protein Analysis

**Figure 5:**
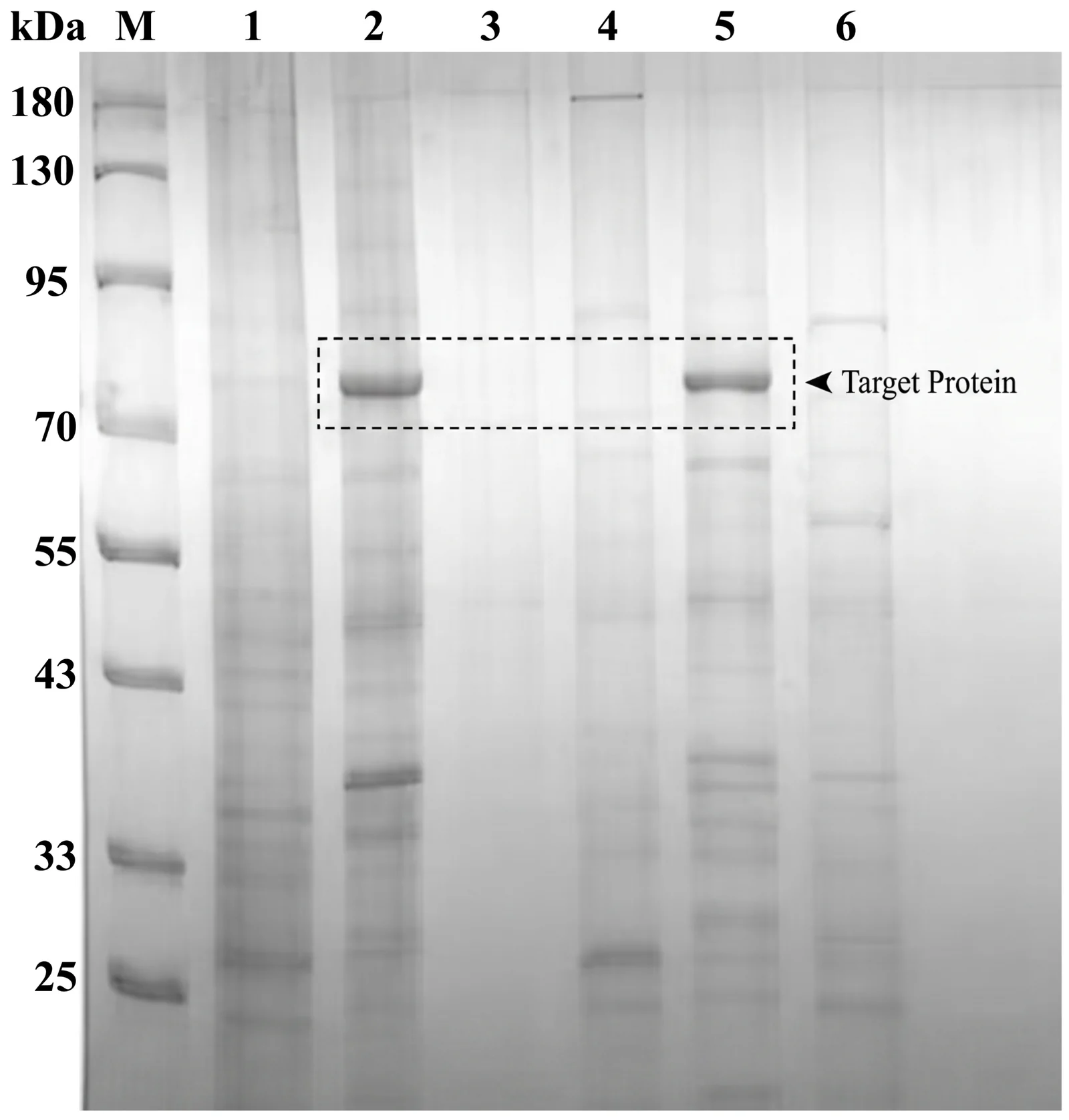
Expression of cry1Ac in *E. coli* BL21(DE3) analyzed by SDS-PAGE. M: Protein Marker; 1: pet28-DE3 blank control; 2: Induced supernatant of LBW8-DE3; 3: Induced precipitate of LBW8-DE3. The target protein (~79 kDa) is mainly present in the precipitate as inclusion bodies.

Following transformation of LBW08-1ac315 into *E. coli* BL21(DE3) and IPTG induction, SDS-PAGE analysis revealed a specific band at approximately 138 kDa, corresponding to the expected molecular weight of the Cry1Ac toxin protein (Figure 5). The target protein accumulated predominantly in the insoluble fraction as inclusion bodies. Densitometric analysis using ImageJ software indicated that the target protein accounted for 92.3% of total protein, meeting the purity requirement for subsequent insecticidal bioassays.

### 3.2. Verification of Integrated Control Efficacy in the Greenhouse

**Figure 6:**
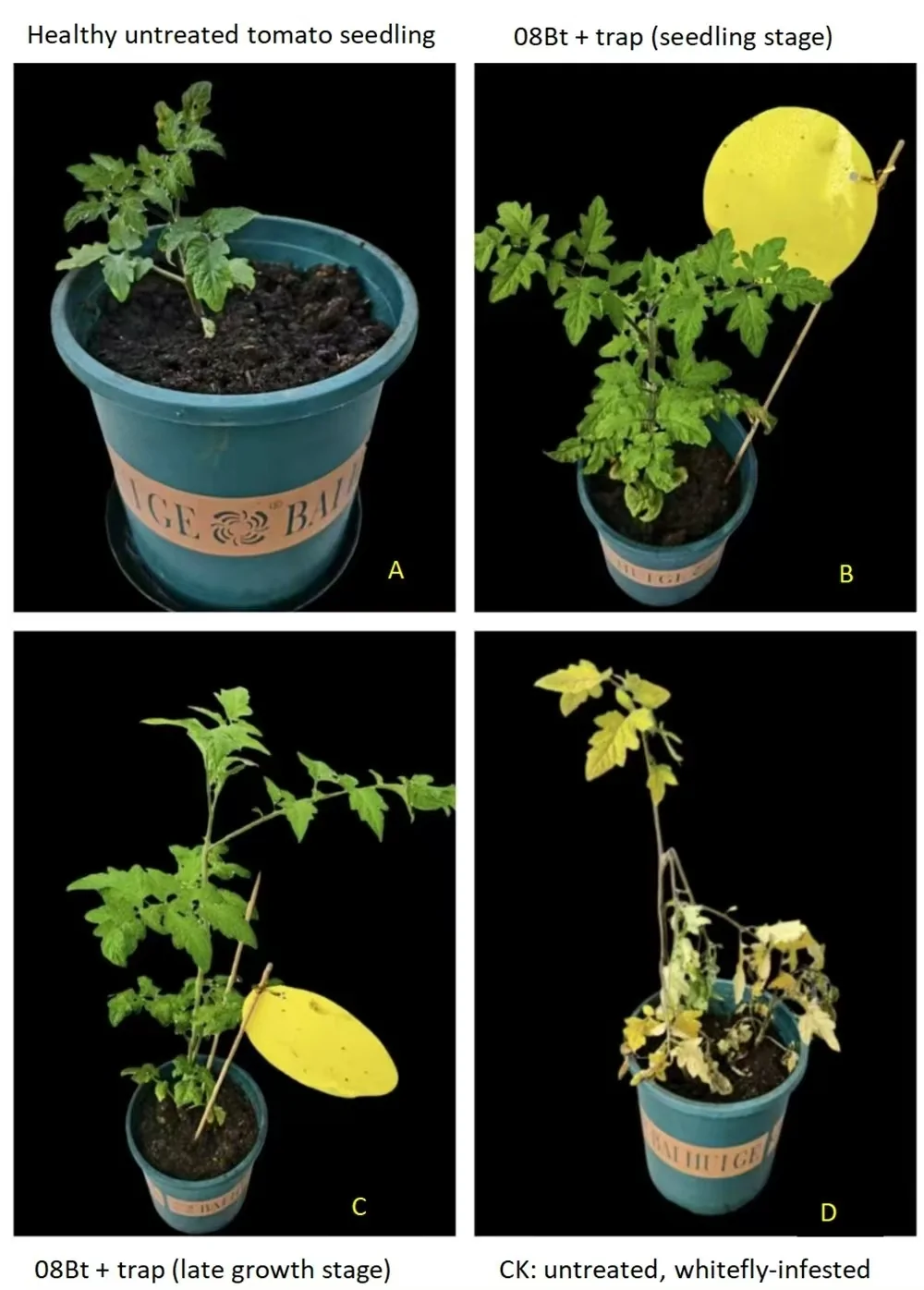
Growth status of tomato plants under different treatments. Note: (**A**): Untreated healthy tomato seedlings; (**B**): Yellow sticky insect board + 08bt treatment group during seedling stage; (**C**): Yellow sticky insect plate + 08bt treatment group in the late growth stage; (**D**): Tomato plants (CK) affected by greenhouse whitefly infestation.

Tomato plants exposed to continuous *Trialeurodes vaporariorum* infestation without intervention (CK) developed pronounced chlorosis, wilting, and necrosis, and growth was severely stunted or ceased entirely (Figure 6D). In contrast, healthy untreated seedlings displayed vigorous growth and well-expanded leaves (Figure 6A). Plants treated with the combination of Bt toxin and yellow sticky traps remained dark green and fully expanded, with significantly greater plant height and branch number compared with the CK group (Figure 6B,C; *p* < 0.05).

**Figure 7:**
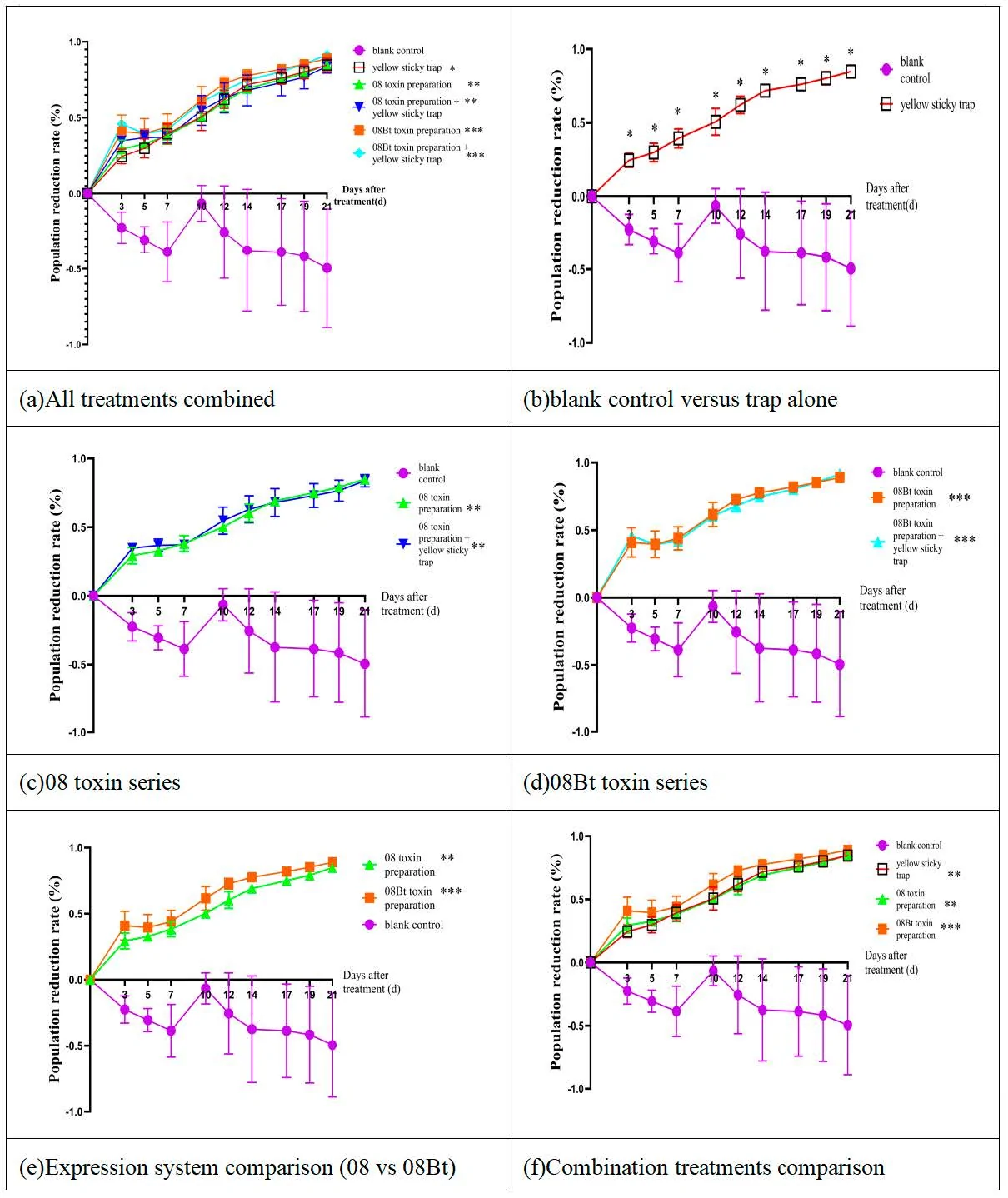
Dynamic changes in the population reduction rate of *Trialeurodes vaporariorum* on tomato plants under different control measures. Data are means ± SE (n = 6). * *p* < 0.05, ** *p* < 0.01, *** *p* < 0.001, indicating statistically significant differences.

**Figure 8:**
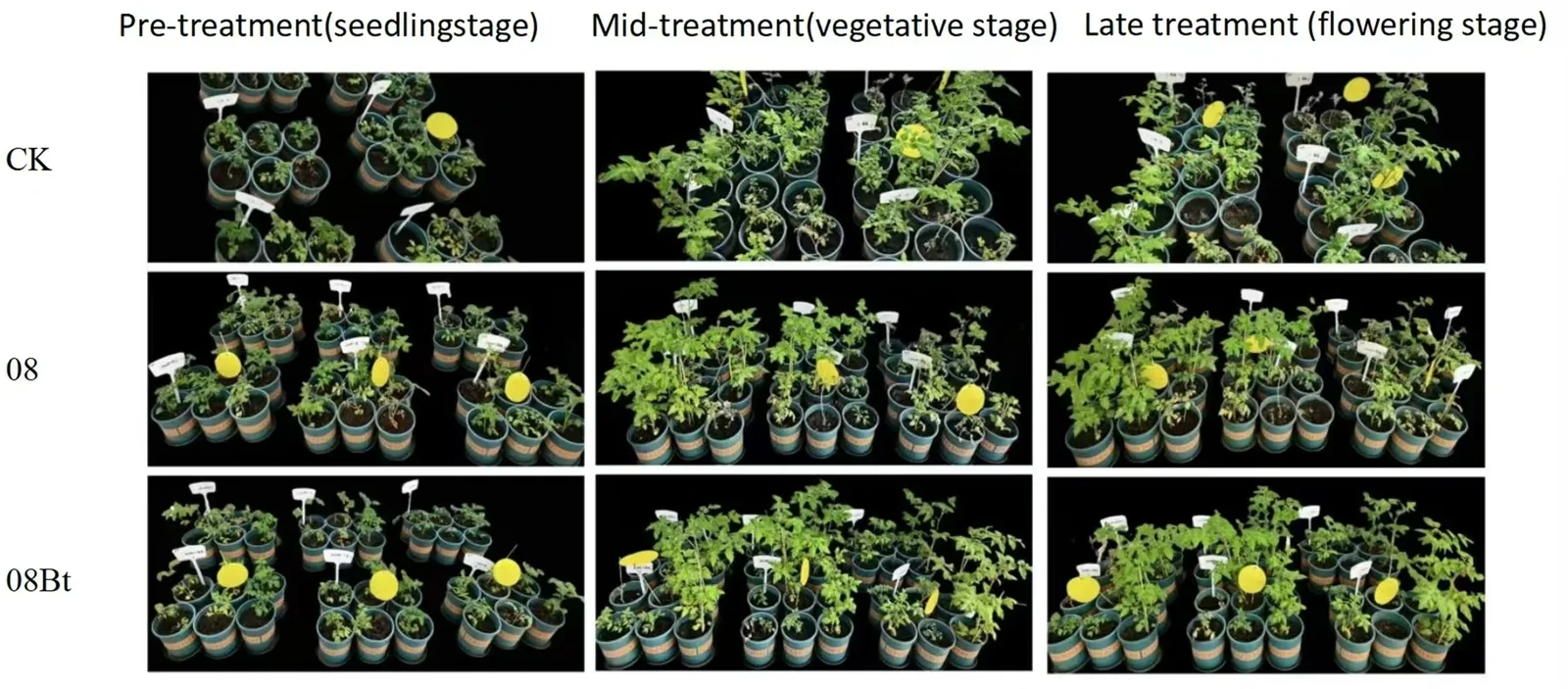
Population growth of tomato plants under different control measures against greenhouse whitefly.

Whitefly population reduction rates increased over time in all treated groups, whereas the untreated control (CK) consistently showed negative values, indicating net population growth (Figure 7). At the plot scale, the uniformity of plant growth varied markedly among treatments. In the untreated control, over 80% of plants were damaged and the majority of leaves were rated at damage levels 3–4. Single applications of yellow sticky traps or Bt toxin reduced the proportion of damaged plants to 45% and 38%, respectively, with most leaves falling into levels 1–2. The combined Bt + trap treatment produced the most uniform stand, with fewer than 10% of plants exhibiting damage and none exceeding level 3. Tomato plants in the combined treatment group maintained continuous growth throughout the experimental period, whereas plant growth in the CK group plateaued or declined after 14 d (Figure 8). It is important to note that the CK and trap-alone treatments received the same concentration of surfactant (0.05% Tween-20) as the Bt-treated groups; thus, any observed reduction in whitefly populations in the Bt treatments is attributable to the toxin protein itself, not to the surfactant.

**Figure 9:**
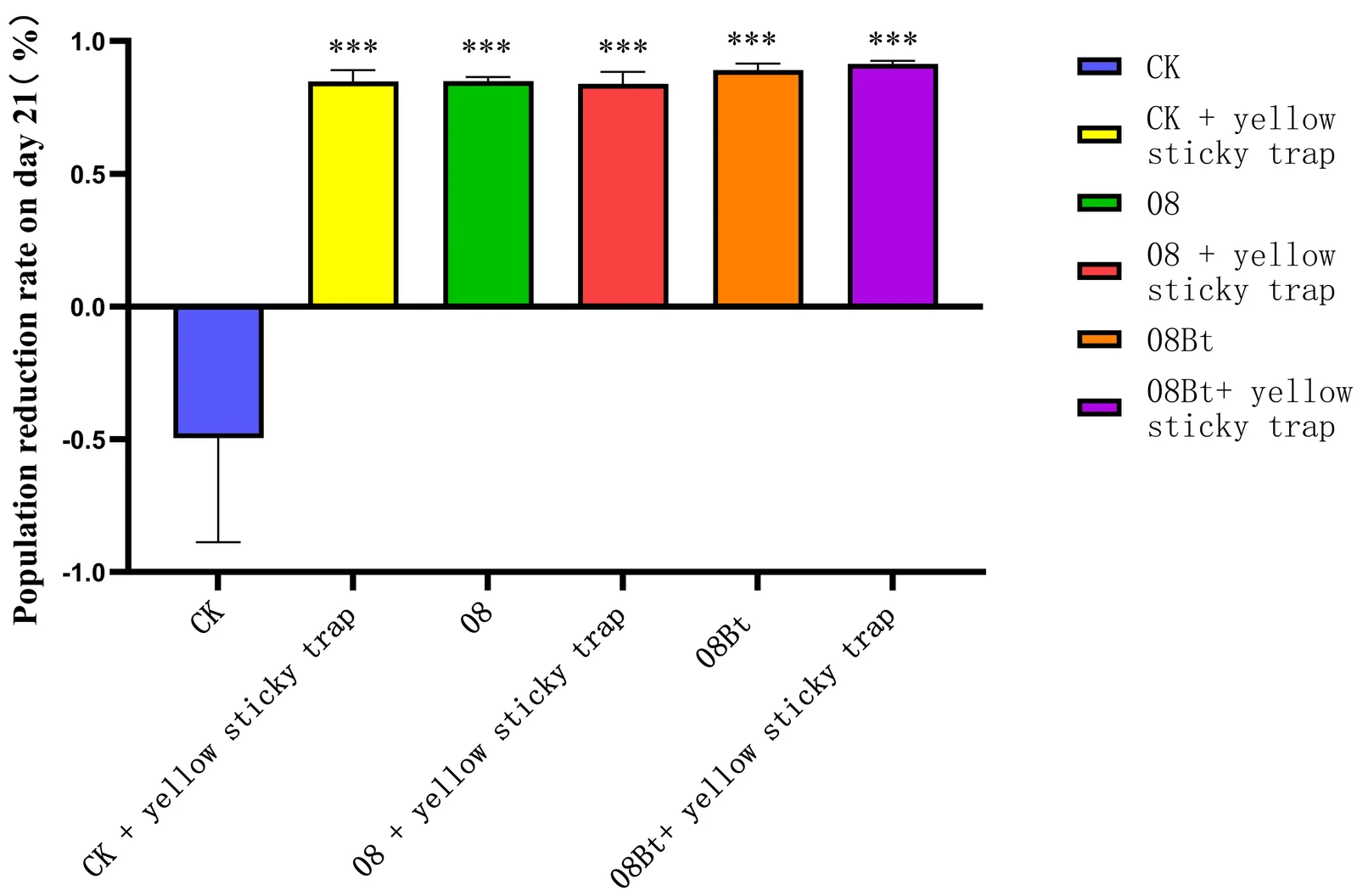
Population reduction rate of *Trialeurodes vaporariorum* on day 21 under different control measures. Data are means ± SE (n = 6). Different lowercase letters indicate significant differences compared with CK (*p* < 0.05); ns indicates no significant differences among treatment groups (Tukey HSD, *p* > 0.05). *** *p* < 0.001, indicating statistically significant differences.

Repeated-measures ANOVA revealed significant main effects of treatment, time, and their interaction (all *p* < 0.0001). The significant treatment × time interaction indicates that the trajectories of population change differed among treatments, confirming that the observed differences cannot be attributed solely to plant decay or other time-related confounding factors. Population reduction reached 55.76% at 3 d and 94.87% at 21 d for the 08Bt + yellow sticky trap combination, demonstrating both rapid and sustained control (Table 5). At 21 d, all treatments significantly reduced whitefly populations compared with CK (*p* < 0.0001; Figure 9). Among the five treatments, the 08Bt + trap combination achieved the highest mean population reduction (94.87%), numerically outperforming all other treatments: 08Bt alone (92.13%), the trap-only treatment (89.64%), the 08 formulation + trap (88.17%), and the 08 formulation alone (86.32%). No pairwise comparison among treatment groups reached statistical significance at any observation time point (Tukey HSD, *p* > 0.05). The addition of yellow sticky traps to the 08Bt formulation resulted in a modest numerical increase in population reduction, whereas the 08 + trap combination did not produce a comparable numerical improvement. Collectively, these results indicate that the JM110-produced 08Bt toxin provides numerically higher control than the 08 formulation, and its combination with yellow sticky traps achieves the highest level of whitefly suppression among the strategies tested.

## 4. Discussions

This study explored the potential of Bt proteins for suppressing *T. vaporariorum* and optimizing synergistic control strategies, a key focus of IPM for protected agriculture [18]. Our results confirm the insecticidal activity of Bt toxin proteins (08 and 08bt) against *T. vaporariorum*, reveal the impact of competent cell background on toxin expression and efficacy, and clarify the synergistic mechanism with yellow sticky traps. These findings offer empirical support for Bt-based integrated control tactics that can be incorporated into broader IPM frameworks for protected agriculture. The following discussion is organized into four thematic subsections:

### 4.1. Expression System Optimization

The 08Bt toxin (plasmid demethylated in JM110 before transformation into HD73^−^) achieved substantially higher purity and insecticidal activity than the 08 formulation (direct transformation without demethylation), consistent with earlier reports. JM110 cells lack *dam* and *dcm* methylase genes and exhibit no restriction endonuclease activity, which protects introduced genes from methylation-dependent degradation and greatly improves heterologous toxin expression efficiency and stability [22]. Conventional *E. coli* competent cells typically show lower expression and are prone to methylation interference, resulting in insufficient synthesis of active toxin and limited pest control [23]. The genotypic background of the host strain is therefore a key factor affecting toxin expression; optimizing the host can markedly enhance toxin activity and yield [23]. The metabolic traits of JM110 are more favorable for high-level Bt toxin expression, highlighting how crucial competent cell selection is for the final efficacy of Bt-based products [24].

### 4.2. Synergy with Physical Control

The 08Bt + trap combination consistently achieved the numerically highest population reduction across all time points. Although the synergistic interaction did not reach statistical significance (Tukey HSD, *p* > 0.05), the trend toward improved control is consistent with the mechanistic complementarity between nymphicidal Bt toxin activity and adult trapping. This discrepancy is closely tied to expression efficiency, active ingredient content, and complementarity across life stages. Synergistic interactions between biopesticides and physical methods depend on mechanistic complementarity and formulation potency [25]: 08Bt effectively kills nymphs while yellow sticky traps strongly attract and capture adults, creating a full-stage control system. Integrating physical tools with biopesticides overcomes the limitations of single-strategy applications and reduces pesticide inputs [16], as demonstrated by the 08Bt + trap combination achieving nearly 95% population reduction. Low-activity formulations often fail to establish meaningful synergy with physical control, explaining the lack of synergy in the 08 + trap group, where insufficient toxin expression led to weak, short-lived nymphicidal activity that could not effectively complement adult trapping. Environmental conditions may also influence insect feeding behavior and biopesticide performance. Synergy between biopesticides and physical methods represents a core direction in green IPM because it reduces reliance on synthetic insecticides and lessens environmental interference with field performance [26]. In our experimental design, the surfactant (Tween-20) was applied at the same concentration to all treatments, including the controls, thereby excluding the possibility that the observed efficacy was due to surfactant effects rather than the Bt toxin itself.

### 4.3. Mechanistic Basis of Bt Activity

Bt toxins are environmentally safe, selective toward non-target organisms, and less prone to resistance development, making them widely applicable in sustainable pest management [1]. Their crystalline protein nature underpins their value as bioinsecticides, supporting the supplement of chemical insecticides and the reduction in agricultural non-point pollution and farmland biodiversity loss [1]. The high-purity expression of 08Bt toxin (≥90%) ensured stable insecticidal activity with minimal interference from impurity proteins, reinforcing the importance of high purity for improving efficacy and lowering environmental risks. Toxin purity correlates positively with insecticidal activity; high purity maximizes toxic effects and facilitates large-scale application [23]. Medium optimization using response surface methodology has also been shown to enhance the purity and yield of Bt toxins, underscoring the value of high-quality toxin preparations for effective control [23].

Bt toxins kill target insects mainly through stomach poisoning, damaging midgut epithelial cells and causing feeding inhibition and metabolic failure [1]. They can specifically bind to midgut epithelial cell receptors and trigger apoptosis [2], consistent with the strong nymphicidal activity of 08Bt observed here. Insecticidal activity is closely related to binding affinity for midgut receptors; higher expression strengthens binding efficiency and improves toxicity [24]. Beyond direct toxicity, Bt toxins can shift midgut microbial communities toward more harmful taxa, further boosting insecticidal activity [24]. This gut microbiota-mediated mechanism has been confirmed in multiple insect–Bt systems: transgenic Bt maize alters gut bacterial communities and host gene expression to enhance mortality in Mythimna separata [24], Bt Cry1Ab/2Ab toxins disrupt the gut bacterial structure of Locusta migratoria through host immune responses [9], and host resistance to Bt has been linked to altered gut bacterial communities [24]. These findings provide a mechanistic explanation for the numerically higher performance of 08bt. Both structural integrity and receptor-binding specificity determine Bt toxin activity. Highly expressed and purified toxins exert stronger stomach poisoning effects [27], consistent with the numerically higher performance of high-purity 08Bt observed in this study.

### 4.4. Limitations and Future Directions

Despite the progress made, the majority of experiments were conducted under controlled greenhouse conditions; field applicability, persistence, and effects on non-target organisms of 08Bt and the combined treatments require verification. Additionally, while our experimental design (randomized complete block design with repeated measures) and analytical approach (repeated-measures ANOVA with percentage reduction metrics) account for temporal trends and control for between-plant variation, we acknowledge that plant condition, particularly the plant decay observed in the untreated control, is inherently linked to pest pressure. This is not a confounder in our design, as we intentionally measure treatment effects relative to a realistic, pest-affected baseline; however, future studies could further disentangle plant-mediated effects through controlled factorial experiments with artificial defoliation or standardized plant damage induction. Future studies should integrate field validation, toxicological analysis, and molecular biology techniques to clarify the specific insecticidal pathway and environmental adaptability of 08bt, optimize its formulation and application, and explore compatibility with other biological control agents such as parasitoids. Crop species can significantly influence Bt toxicity and residual activity, so field validation is indispensable for assessing practical performance before large-scale deployment [28]. Insights from management programs for major pests like fall armyworm and emerging tools such as RNA interference [29] may provide additional technical resources for greenhouse whitefly control. Addressing these gaps will strengthen the application of Bt toxins against piercing-sucking pests, refine green IPM strategies, and advance sustainable agricultural pest management. The integrated control tactic explored here (Bt toxin plus traps) should be viewed as one promising element of comprehensive IPM programs, rather than a complete IPM system itself.

While our corrected efficacy estimates robustly demonstrate the relative performance of different treatments, they do not capture age-specific effects or potential sublethal impacts on reproduction and development. However, for the purposes of this applied efficacy trial, which was designed to evaluate practical greenhouse control performance—the repeated-measures ANOVA and population reduction metrics used here are appropriate and sufficient. We also note that all treatments were assessed under identical conditions, ensuring that comparisons among treatments are internally valid. Future work will employ life-table analyses to quantify the demographic impact of 08Bt toxin on *T. vaporariorum* populations, which will further clarify the mechanisms underlying the observed synergy and help refine application schedules.

Additionally, while yellow sticky traps are effective for monitoring and mass-trapping adults, their use is not without ecological drawbacks. Sticky traps are non-selective and can capture a wide range of non-target arthropods, including natural enemies such as parasitoids and predators that are essential for biological control of whiteflies and other greenhouse pests. Excessive or poorly timed deployment of sticky traps may reduce the abundance and effectiveness of these beneficial insects, thereby undermining the long-term sustainability of integrated pest management (IPM) programs. For example, Shi et al. [30] demonstrated that yellow sticky cards significantly reduce the abundance of natural enemies in tea plantations, and Gan et al. [31] reported that yellow sticky cards reduce the numbers of Trichogramma parasitoids in pear orchards. Similar negative impacts on predatory mites and other generalist predators have been documented in other cropping systems [32,33]. Furthermore, the use of yellow sticky traps can also negatively affect pollinators such as bumblebees and hoverflies, which are commonly used for pollination in greenhouse tomato production [34,35]. Therefore, although the 08Bt + trap combination showed high numerical efficacy in our greenhouse trial, growers should weigh the trade-off between immediate whitefly suppression and potential disruption of biological control services. Future IPM strategies should consider using traps only during critical infestation periods, employing trap-free refuges for natural enemies, or combining traps with selective biopesticides that spare beneficial fauna. Our results should be interpreted with this ecological caveat in mind, and further field studies are needed to quantify the net effect of trap use on the overall predator-prey balance in tomato greenhouses.

### 4.5. Implications for Decision-Support Systems and IPM Implementation

The temporal efficacy data presented in Table 5 and Figure 7 form a critical input for DSS models targeting *T. vaporariorum*. A functional DSS for greenhouse pest management typically integrates multiple criteria: pest population dynamics, control measure efficacy, application costs, labor, and crop value [18,25]. Our results indicate that the 08Bt + trap combination consistently outperformed other treatments in terms of peak and sustained population reduction (94.87% at 21 d), but this superior efficacy must be weighed against the additional costs of Bt production and application. In contrast, the trap-only treatment achieved 89.64% reduction at a lower input cost, making it a highly cost-effective baseline strategy. By providing these quantitative trade-offs, our study offers a preliminary decision matrix for growers. For instance, under low to moderate whitefly pressure, the trap-only strategy may suffice; under high pressure or when virus transmission risk is elevated, the incremental benefit of adding 08Bt (around 5% additional suppression) might justify the extra investment. Future DSS development should incorporate economic injury levels (EILs) and action thresholds, as well as environmental variables such as temperature and humidity, which influence both Bt persistence and whitefly development rates [26,28]. This DSS-oriented interpretation aligns our experimental findings with practical farm-level decision-making, thereby bridging the gap between academic research and on-farm pest management.

## 5. Conclusions

This study evaluated the insecticidal activity of Bt toxin proteins (08 and 08Bt) against *T. vaporariorum* under greenhouse conditions and characterized how different expression systems affect toxin production. The 08Bt toxin, produced via the HD73-expression system, accumulated at a higher concentration and achieved a purity of 92.3%. Under the tested conditions, all active treatments significantly reduced whitefly populations compared with the untreated control at 21 d. The 08Bt + trap combination showed a numerically higher population reduction (94.87%) than the other active treatments (86.32–92.13%). However, the differences among the active treatment groups were not statistically significant. These data indicate that foliar application of Bt toxins provides no detectable additional control efficacy against *T. vaporariorum* beyond the effect of yellow sticky traps alone. Yellow sticky traps deliver equivalent control performance at lower input cost. We therefore propose a DSS-guided approach: yellow sticky traps alone provide a highly cost-effective baseline management tactic under most greenhouse scenarios. The incremental benefit of adding Bt toxin should be considered only when pest pressure exceeds critical economic thresholds or when protecting high-value crops. By quantifying the dynamic efficacy and cost-effectiveness of each strategy, this study provides essential parameters for a DSS framework, enabling growers to make evidence-based, context-specific management decisions. Our findings thus contribute to the rationalization of IPM inputs, reducing unnecessary biopesticide use while maintaining effective pest suppression in protected agriculture.

## Figures and Tables

**Table 1:** PCR reaction system.

Component	Volume (μL)
Template DNA (100 ng/μL)	1.0
2× Magic Green Taq SuperMix	12.5
Forward primer (10 μM)	1.0
Reverse primer (10 μM)	1.0
Sterile double-distilled water (ddH_2_O)	9.5
Total reaction volume	25

**Table 2:** PCR reaction program.

Program	Temperature	Time
Pre-denaturation	95 °C	5 min
Denaturation	95 °C	1 min
Annealing	50 °C	1 min
Extension	72 °C	30 s/kb
Final extension	70 °C	10 min
Preservation	4 °C	∞

Note: ∞ represents indefinite preservation; 30 s/kb means the extension time is set as 30 s per kilobase of the target fragment length.

**Table 3:** Carrier digestion reaction system.

Purified carrier	7 μL
rCustSmart Buffer	2 μL
Dpn1	1 μL

**Table 4:** Seamless cloning reaction system.

Component	Volume
2× Ezmax Ultra Universal CloneMix	5 μL
linearized vector	20 ng/kb (Minimum 40 ng)
Exogenous DNA insertion fragment	40 ng/kb (Minimum 20 ng/segment)
Sterile ddH_2_O	Up to 10 μL

**Table 5:** Correction and control effects of different treatments on greenhouse whiteflies.

Treatment Group	Initial Insect Population (Average)	Processed for 3 Days (72 h)	Processed for 5 Days	Processed for 7 Days	Processed for 10 Days	Processed for 12 Days	Processed for 14 Days	Processed for 17 Days	Processed for 19 Days	Processed for 21 Days
Blank control (CK)	56.33	−22.59%	−30.65%	−38.69%	−6.59%	−25.60%	−37.56%	−38.73%	−41.66%	−49.53%
Yellow sticky insect board	71.89	38.42%	43.17%	56.29%	67.35%	76.12%	82.05%	84.78%	87.21%	89.64%
08 formulation	88.56	42.36%	46.89%	51.23%	58.76%	65.41%	72.33%	78.56%	82.14%	86.32%
08 formulation + yellow board	87.56	46.91%	49.25%	52.78%	61.34%	68.92%	74.56%	79.12%	83.45%	88.17%
08bt toxic protein	85.06	52.09%	57.82%	62.45%	74.12%	80.34%	84.67%	87.92%	90.56%	92.13%
08bt + yellow board	73.11	55.76%	60.15%	64.78%	77.56%	83.89%	89.23%	92.45%	94.12%	94.87%

Note: Values for nymphs are corrected mortality rates; values for adults are reduction rates relative to no-trap controls. No significant pairwise differences were found among treatments at any time point (Tukey HSD, *p* > 0.05).

## Data Availability

The data presented in this study are openly available in https://doi.org/10.6084/m9.figshare.32390331.
